# Integration of Design of Experiments, Analysis of Variance and Response Surface Methodology in Assessing Heterogeneous Catalysts Processes: A Minireview

**DOI:** 10.1002/open.202400148

**Published:** 2024-12-16

**Authors:** Carlos Esteban Aristizábal‐Alzate, Eva Castillejos‐López, Ana Belén Dongil, Manuel Romero‐Sáez

**Affiliations:** ^1^ Grupo Química Básica Aplicada y Ambiente–Alquimia Facultad de Ciencias Exactas y Aplicadas Instituto Tecnológico Metropolitano Robledo, Medellín 050034 Colombia; ^2^ Department of Inorganic and Technical Chemistry Faculty of Sciences, UNED Madrid 28232 Spain; ^3^ Grupo de Química Verde y Catálisis Instituto de Catálisis y Petroleoquímica CSIC Cantoblanco, Madrid 28049 España

**Keywords:** Experimental design, Heterogeneous catalyst, ANOVA, Catalyst assessment, Response surface methodology

## Abstract

This work reviews the application of Analysis of Variance (ANOVA), Design of Experiments (DOE), and Response Surface Methodology (RSM) in heterogeneous catalysis, based on an analysis of recent scientific literature. Heterogeneous catalysis plays a key role in various industries, guiding chemical reactions, optimizing the performance of different processes and/or prioritizing products, and ANOVA, DOE and RSM are valuable tools to understand the intricate relationships between catalyst properties, process variables and reaction responses. This understanding facilitates improvements in catalysts performance and selectivity, thereby optimizing processes. Drawing insights from recent studies, this minireview highlights the different applications of ANOVA‐based experimental designs and RSM optimization techniques in catalytic process evaluation. Therefore, this review provides valuable insights into these current statistical methodologies applied to heterogeneous catalysis research, informing the relevance and future directions of the research in the field.

## Graphical abstract

This review highlights how the integration of experimental design, variance analysis, and response surface methodology contributes to the advancement of heterogeneous catalysis. The synergistic application of these methodologies allows to reveal critical insights into different processes, optimize catalytic efficiency, and make data‐driven adjustments to catalyst synthesis and reaction conditions, which further lays the foundation for developing precise and sustainable catalytic processes.

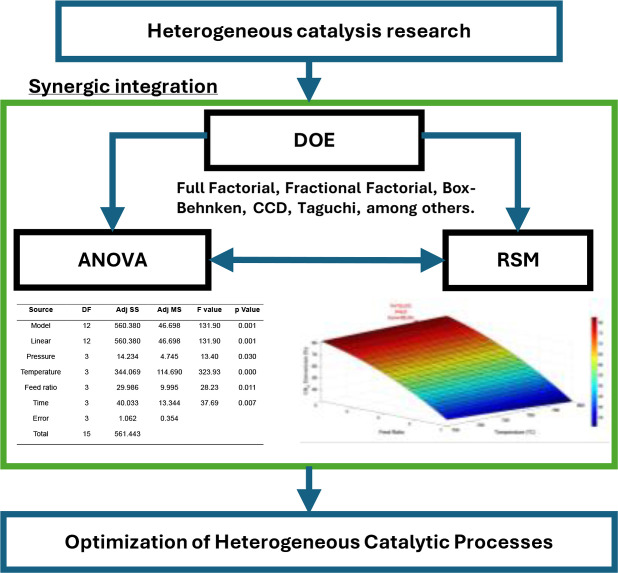

## 1 Introduction

Statistical experimental design and principal component analysis are well‐recognized methodologies to deploy quality by design in both research and industrial sectors.^[1]^ While their application in research may not directly result in the novel catalysts discovery, they significantly enhance the understanding of how process parameters influence catalytic performance.^[2]^ Furthermore, employing these methodologies provides a deeper insight into the correlations among diverse performance indicators and it can optimize the number of experiments.[^2^, ^3^, ^4^]

Catalysis is a very important field, since over 85 % of industrial processes employ a catalyst in at least one of their processes, with heterogeneous catalysis being used in up to 80 % of these catalytic processes.^[5]^ A variety of catalytic materials of different chemical nature are available as bulk or as supported materials. Recently, it has become apparent that for catalysis, in particular for complex reactions, high selectivity for a product is demanded, and active sites on the nanoscale are required. Typical materials for such nanoscale active sites are nanoparticles not exceeding a certain size, as well as nanoscale fibers, nanotubes and nanostructured porous materials, such as zeolites. Such materials may consist of metals, metal alloys, skeletal metal structures, reducible (redox‐type) and irreducible metal oxides of acidic and basic nature, metal carbides, and metal–organic frameworks. When these materials are used in a specific chemical reaction, the main requirements are usually activity and selectivity, which can be affected by transport limitations.^[6]^

The number of variables that can influence the catalytic performance is high. Parameters related to the catalyst such as preparation methods, active phase, particle size and shape, exposed planes, among others can drastically change the catalytic performance. In addition, the operation parameters such as pressure, temperature or feed concentration have to be considered.[^2^, ^7^] Despite the knowledge of the factors affecting catalytic performance, heterogeneous catalysis persists as an empirical science, primarily owing to the intricate nature of the surface reactions involved.^[7]^

Therefore, incorporating design of experiments (DOE) into catalyst development processes improves the prospects of discovering new routes for advancement.[^2^, ^8^] Additionally, these methodologies are a relevant approach in chemical research, since they consider all the parameters of the catalyst development life cycle based on the desired or expected results, which facilitates the determination of the operating conditions necessary for process scaling.^[9]^

For reactive systems, it is essential to determine the factors to be examined, establish the experimental range for each factor, define the variation degrees (referred to as levels) for these factors, and choose an appropriate experimental design methodology (e. g., Box‐Behnken, full factorial, Doehlert matrix, Taguchi, etc.).[^10^, ^11^] The Box‐Behnken design (BBD) is widely recognized as the predominant design within response surface methodology (RSM) owing to its efficiency and cost‐effectiveness in optimizing the parameters of chemical processes.^[12]^ On the other hand, J.H. Türkcan, et al.^[13]^ report that Taguchi is a powerful and unique method that allows optimization with fewer experiments, and it helps to save a lot of time and effort in experimental work.

**Figure 1:**
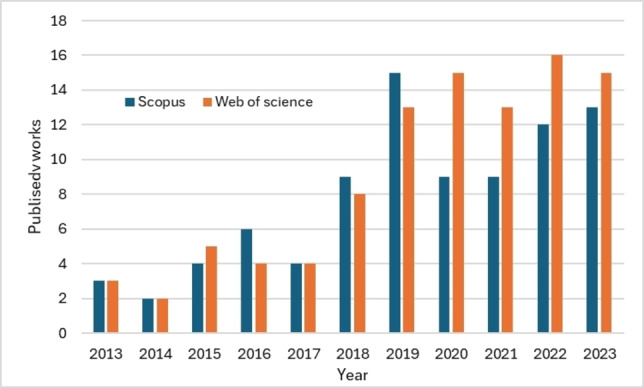
Published documents per year on Scopus and Web of Science databases (2013–2023).

Although it is a field of great importance, there are still not many works that integrate catalytic results and statistical analysis. Figure 1 shows the number of published documents per year (2013–2023) related to the heterogeneous catalysis processes and their integration with statistical analysis on Scopus and Web of Science.

According to Figure 1, the research on the topic shows a sparse number of publications averaging fewer than six per year between 2013–2017, and sixteen each year for 2018–2023 period. However, in recent years, the number of works in the field shows an increasing trend, highlighting the importance of integrating these techniques with experimental procedures. This integration aims to increase the rigor of the obtained results and to determine, with statistical and quantitative significance, the influence of the experimental process variables on a response variable.

Therefore, the current minireview provides a relevant contribution to the application of these statistical techniques into heterogeneous catalyst research and catalytic process development, due to a significant obstacle lies in the “language barrier” among researchers from diverse disciplines. Notably, empirical (data‐driven) methods present challenges, as they are typically performed by statisticians rather than chemists or chemical engineers.^[2]^

## 2 Integration of Experimental Procedure and Modelling Methods in Catalyst Research

**Figure 2:**
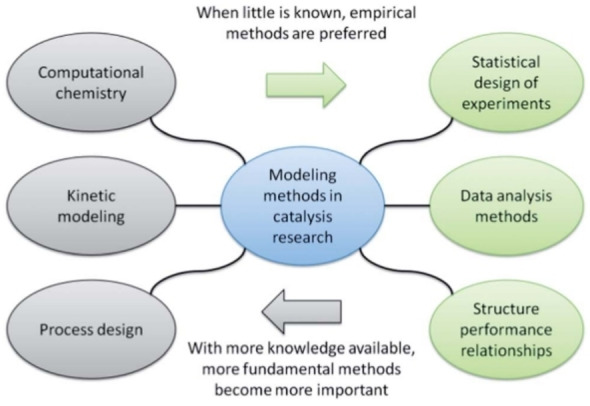
Modeling methods used in catalysis research. Reproduced from E. J. Ras, G. Rothenberg^[2]^ with permission from the Royal Society of Chemistry.

Modeling methods in catalysis can be classified into two groups, fundamental and empirical,^[2]^ as illustrated in Figure 2. The first group comprising computational chemistry, kinetic modeling, and reactor design, emphasizes understanding reaction mechanisms and applying engineering principles. These methods are helpful when mechanistic insights or reactor constraints are known in advance. The second group consists of data‐driven models, which do not rely on assumptions regarding reaction mechanisms or reactor configurations. Consequently, they are suitable for early‐stage research with limited information.

Contrary to common perception, the most effective approach involves the integration of modeling and experimental research methods in catalysis,[^2^, ^14^] implementing a synergistic collaboration between teams from these two fields of science. However, there are already reports in catalyst research using these techniques. Thus, reports and research have been found on biodiesel production using used cooking oil[^15^, ^16^, ^17^] and oil from first‐generation biomass,[^8^, ^10^, ^18^, ^19^, ^20^] biogas and organic compounds reforming,[^4^, ^9^, ^21^, ^22^, ^23^] organic pollutant remotion,[^14^, ^24^, ^25^, ^26^, ^27^, ^28^, ^29^] other high added value chemical synthesis[^10^, ^12^, ^30^, ^31^, ^32^, ^33^, ^34^] and for optimization and evaluation of different chemical process via simulation.[^31^, ^35^]

The integration of computational chemistry and kinetic studies with statistical modeling methods provides a comprehensive framework for optimizing catalytic processes. Computational approaches in catalyst research help to identify critical reaction conditions and mechanistic insights that can be incorporated into DOE and then analyzed by ANOVA for efficient experimental variables exploration. This was confirmed by several studies, such as methanol synthesis from coffee pulp gasification,^[35]^ heterogeneous catalytic oligomerization optimization of 1‐butene into light olefins,^[10]^ among others. Meanwhile, kinetic studies play a crucial role by providing reaction rate and activation energy data, which can be used to refine models and improve process optimization via RSM, as can be observed in the study of 4‐aminoantipyrine synthesis.^[36]^ This combination of computational and experimental approaches enables more efficient catalyst design by reducing the number of experimental runs required and improving the accuracy of statistical models, as observed in the oxidative dehydrogenation of propane (ODHP) over a vanadium–graphene catalyst^[37]^ and catalytic CO_2_ methanation.^[38]^ The integration of these techniques enables a solid understanding of catalytic mechanisms, and accelerates the advancement of the field of catalytic materials and efficient chemical processes for industrial applications.^[6]^

Furthermore, the employment of these methods in heterogeneous catalyst research requires the use of software for data analysis and statistical development to integrate the experimental results with math modeling. Some reported software in heterogeneous catalyst statistical assessment are MATLAB,[^15^, ^35^] Statistica,[^10^, ^39^] Design Expert Software,[^8^, ^24^, ^37^, ^40^, ^41^, ^42^] MINITAB[^18^, ^25^, ^43^] and SPSS.^[34]^ Nevertheless, software selection does not influence the data analysis since the math and statistical fundamentals are the same.

## 3 Design of Experiments

Statistical experimental design allows researchers to efficiently explore the effects of multiple variables on reaction outcomes by systematically varying experimental factors while minimizing the number of experiments required. The employment of these techniques such as factorial design, response surface methodology, and design of experiments (DOE) allow researchers to identify key factors that influence catalytic performance and to elucidate optimal reaction conditions. By randomizing experimental conditions, any potential confounding influences are mitigated, allowing for the establishment of causal links between experimental factors and the observed values of measured variables.[^15^, ^44^] Furthermore, statistical methods offer certain advantages over experimental studies, as they demand less time and funding resources compared to their experimental counterparts.[^9^, ^11^, ^40^]

DOE method is commonly categorized into two main approaches: Full Factorial Design (FFD) and Fractional Factorial Design, also known as Taguchi Experimental Design (TED). In FFD, all possible combinations of parameter levels are tested to analyze the results comprehensively. Conversely, using TED, only a selected subset of the levels is utilized in the analysis, allowing for a more efficient use of resources.^[45]^ Nevertheless, the DOE type should be carefully chosen based on the context and case study to ensure that meaningful data is collected, without wasting time or resources unnecessarily.[^13^, ^36^]

A conventional experimental design process encompasses three primary phases: (1) factor screening, (2) optimization, and (3) robustness testing. In factor screening phase, numerous variables are examined through a limited number of experiments. The aim is to identify and eliminate variables with minimal impact on performance. Only the pertinent variables proceed to the optimization phase (2), which establishes quantitative relationships between variables and responses. The subsequent robustness testing phase (3) involves sensitivity analysis, assessing the anticipated stability of the optimized system.^[2]^ Experimental designs suitable for first‐order models, such as factorial design, are applicable when the dataset lacks curvature. However, if the response function cannot be adequately described by linear functions and exhibits curvature, alternative designs tailored for quadratic response surfaces should be employed. These include factorial design, Box‐Behnken design, central composite design (CCD), Doehlert design, and Taguchi approach, among others.[^24^, ^39^, ^46^]

DOE considers levels for the selected explanatory variables or factors, which are the values to be evaluated and to determine their influence on the response variable. For example, the selection of these variables and their ranges was obtained by the established operating parameters of the commercial catalytic process, as well as the feasibility of the catalytic reactor setup or carefully examining the limits within which the experiment will be conducted.[^10^, ^11^, ^16^] Another selection tool should be based on the researcher's experience and literature reports to find or to understand the catalyst and the process performance with the most important operational parameters.[^16^, ^39^]

Table 1 shows the response variables, factors, levels, and DOE methodologies for some heterogeneous catalyst and process assessments. Additionally, it illustrates the aim of the research related to the use of these statistical techniques. The inclusion of the works depicted is predicated upon the prevalence of topics within the domain of heterogeneous catalysis and ANOVA, prioritizing research that delves beyond mere operating and reaction variables to encompass a broader spectrum of influential factors.

**Table 1:** Catalyst research development using design of experiment (DOE).

Aim	Response variable	Factor	Levels	DOE methodology	Ref.
To optimize the biodiesel production with respect to the yield of fatty acid methyl Esters, using CaO as catalyst	Fatty Acid Methyl Ester yield (%)	Temperature (°C) Time (min) Methanol to oil molar ratio (mol/mol)	60, 65 and 70 °C 180, 240 and 300 min 12, 15 and 18 mol/mol	Full 2^4^ factorial design with four central points	Latchubuga, C. S, et al.^[39]^
To optimize the reaction variables: methanol/oil molar ratio, reaction time and amount of catalyst for production of biodiesel from palm oil using KF/ZnO catalyst	Biodiesel yield (%)	Catalyst amount (wt %) Reaction time (h) Methanol to oil molar ratio (mol/mol)	2.22 and 5.78 (wt %) 7.22 and 10.78 (h) 7.03 and 12.98 (mol/mol)	2^3^ full factorial central composite design CCD for the three variables, consisting of 8 factorial points, 6 axial points and 6 replicates at the center points	Hameed, B, et al.^[40]^
Modelling and experimental studies focusing on dry reforming of CH_4_ over Ni−Cu/Al_2_O_3_ catalyst coatings.	CH_4_ conversion CO_2_ conversion Catalyst deactivation H_2_:CO ratio	Deposition time (min) Cu/Ni surface area percentage (%) Reaction temperature (°C)	1, 3 and 5 min 10, 20 and 30 % 700, 750 and 800 °C	Box–Behnken design (BBD) with three levels	Rezaei, R., Moradi, G., Sharifnia, S.^[22]^
To optimize and kinetics assessment of the methanolysis reaction employing the PKSB catalyst, with the objective of formulating statistical and kinetic models delineating the correlation between the content of fatty acid methyl esters and the operational parameters.	FAME content in the sunflower oil methanolysis	Temperature (°C) Methanol‐to‐oil molar ratio (mol/mol) Catalyst amount (%)	45, 55 and 65 °C 9, 12 and 15 (mol/mol) 3, 5 and 7 (%)	3^3^ full factorial design of experiment (DOE) with five central points	Kostić, M. D, et al.^[11]^
To optimize the process parameters essential for converting low‐cost waste cooking oil into methyl esters, leveraging a cost‐effective source of heterogeneous catalyst derived from eggshells.	The yield of WCO methyl esters	Catalyst calcination temperature (°C) Catalyst calcination time (min) Catalyst loading (wt.%) Alcohol: oil ratio Reaction temperature (°C) Reaction time (min)	700, 800 and 900 °C 60, 90 and 120 min 5, 10 and 15 wt. % 8, 9 and 10 55, 65 and 75 °C 60, 80 and 100 min	The L27 orthogonal array design by Taguchi comprises a 3‐level‐6‐factor array	Singh, T. S., Verma, T. N.^[16]^
To investigate the impact of operational parameters on the heterogeneous Fenton‐type oxidation of RY15.	RY15 color removal (%)	pH of the reaction Contact time (min) Catalyst concentration (g/25 mL)	2 and 6 60 and 120 min 0.1 and 0.3 g/25 mL	A 2^3^ full factorial design (with two repetitions)	Y. Asci, et al.^[25]^
To investigate the impact of gasification and reaction operational parameters on the catalytic methanol synthesis.	Methanol production per reactor tube (mol s^−1^)	Gasification temperature (K) Steam‐biomass ratio Reaction temperature (K)	973.15 and 1073.15 K 0.5 and 1.0 453, 498, 523 and 553 K	A 2^2.^4 factorial design	C. E. Aristizábal‐ Alzate, et al.^[35]^
To determine the main parameters of the production process of the hydrogen from sodium borohydride, using Ni−B catalysts	Hydrogen generation rate (L min^−1^ g_cat_^−1^)	Temperature (K) pH Reducing agent concentration (NaBH_4_/water) Reduction rate (ml dk^−1^)	278, 293, 303 and 318 K 1, 2, 4 and 6 100, 10 and 25 5, 10, 15 and 20	L16 orthogonal array (4^4^) by Taguchi design comprises a 4‐level‐4‐factor array	J. H. Türkcan, et al.^[13]^
To apply design of experiment (DOE) coupled with the artificial neural networks (ANN) in kinetic study of oxidative dehydrogenation of propane (ODHP) to produce light olefins over a vanadium‐graphene catalyst.	Propane conversion (%) Propylene selectivity (%) Ethylene selectivity (%) CO_x_ selectivity (%),	Temperature (°C) Propane/air ratio Feed flowrate ( min^−1^)	400, 450 and 500 (°C) 0.2, 0.6 and 0.8 60, 90 and 180 (ml min^−1^)	CCD with three factors with a total of 30 experiments	M. Fattahi, et al.^[37]^
To evaluate the catalytic wet peroxide oxidation performance of synthesized ferrite by degradation of tartrazine in aqueous solution.	Degradation efficiency of tartrazine (%)	Magnesium ferrite dosage (g L^−1^) Tartrazine concentration (mg L^−1^) H_2_O_2_ concentration (mM)	0.04, 0.08 and 0.12 30, 40 and 50 3.528, 5.292 and 7.056	Box Behnken statistical design for three factors at three levels	A. Soufi, et al.^[29]^
To remove xylene vapor pollutant from the air using new hybrid substrates of TiO_2_‐WO_3_ nanoparticles immobilized on the ZSM‐5 zeolite under UV radiation at ambient temperature.	Removal xylene efficiency (%)	Catalyst concentration (wt %) Xylene concentration (ppm) Flow rate (L min^−1^)	1, 3 and 5 50, 100 and 150 0.3 and 0.5	Taguchi L18 (6^1.^3^3^) orthogonal array design	H. A. Rangkooy, et al.^[26]^

According to Table 1, the factors are not only limited to operational parameters of the catalytic reaction but also include parameters related to catalyst physicochemical properties and its preparation methods. Additionally, the response variable is usually selected among the conversion, yield, selectivity, or amount of the products of interest to be synthesized.[^2^, ^16^, ^21^, ^35^, ^39^] The common aim in reported works is to find values for all explanatory variables that optimize the studied response variable considering the evaluated levels. The number of the explanatory variables considered as factor and response variables in each work depends on the interest in correlating them and to establish math models that can predict the behavior of response variables according to the variations of factor values. On the other hand, it can report and analyze several response variables, as can be seen in R. Rezaei, et al.,^[22]^ which shows independent analysis and results for CH_4_ conversion, CO_2_ conversion, catalyst deactivation and H_2_:CO ratio, response variables considered.

For mathematical modeling and data analysis, DOE method is widely used to apply a numerical coded value for the selected factors or explanatory variables. The coded values assignation depends on the number of levels and usually, reports have a central, maximum and minimum value with equal spacing in the assigned value.[^10^, ^15^, ^39^, ^40^] Additionally, the experiments have to be performed in a completely random order to minimize errors.^[8]^ Table 2 shows examples of the real value of the variable and coded for the factors or experimental variables in some research.

**Table 2:** Experimental variables in coded and actual units for several.

Factor	Coded name	Actual values	Coded values	Reference
Methanol/oil molar ratio	X_1_	6 : 1, 9 : 1 and 12 : 1	1, 2 and 3	N. S. El‐Gendy, S. F. Deriase.^[15]^
Temperature (°C)	A	180, 240 and 300 °C	−1, 0 and 1	C. S. Latchubugata, et al.^[39]^
pH	A	2, 4, 6, 8 and 10	−1.5, −1, 0, 1 and 1.5	F. Shahrezaei, et al.^[24]^
Cu/Ni surface area percentage (%)	X_2_	10, 20 and 30	−1, 0 and 1	R. Rezaei, G. Moradi, S. Sharifnia.^[22]^
Type of catalyst	X_1_	LaFeNaY, LaFeClay_M_ and LaFeZSM5	−1, 0 and 1	Assila, et al.^[47]^

Factors are typically denoted by capital letters (e. g., A, B, C…) or X_i_ (i=0, 1… n), with their lower and higher levels indicated by symbols such as (−) or (−1) and (+) or (+1), respectively. If a middle level is present, it is represented as (0).[^1^, ^25^, ^39^] The regression model devised for predicting the response value has the flexibility to utilize either actual or coded values.^[22]^ Nevertheless, when DOE involves a factor with multiple levels that cannot be adequately described using numerical values, the adoption of coded values becomes imperative for constructing and applying the model. This necessity arises in situations such as when DOE incorporates a factor like the type of catalyst, as exemplified in O. Assila, et al.^[47]^

## 4 Analysis of Variance

Analysis of Variance (ANOVA) is a statistical method used to analyze the differences between two or more groups or treatments in an experiment, evaluating whether the means of these groups are significantly different from each other. ANOVA works by partitioning the total variation observed in a dataset into different sources, such as the variation between groups and the variation within groups. It is a powerful method for determining the contribution of each factor and the significance of the optimization model. Furthermore, ANOVA is conducted to underscore the validity and significance of the experimental data derived from the investigations concerning the influence of the process parameters.^[48]^

ANOVA calculates the F‐value (Fischer's test value) and the sum of squares to evaluate the significance of parameters. A p‐value below 0.05, corresponding to a 5 % significance level or a 95 % confidence interval, indicates statistical significance.[^8^, ^14^] This value of confidence is the most used in the literature.[^8^, ^21^, ^44^] In ANOVA, the increased significance of explanatory variables is evidenced by higher F‐values, also known as F‐statistics, and lower p‐values, commonly referred to as significance probability.[^42^, ^49^] Table 3 shows the ANOVA for biodiesel production from waste cooking oil using S‐TiO_2_/SBA‐15 heterogeneous acid catalyst done by M.N. Hossain, et al.^[48]^

**Table 3:** ANOVA for biodiesel yield evaluating four factors.^[48]^

Source	DF	Adj SS	Adj MS	F value	p Value
Model	12	560.380	46.698	131.90	0.001
Linear	12	560.380	46.698	131.90	0.001
Catalyst (%)	3	14.234	4.745	13.40	0.030
Temperature	3	344.069	114.690	323.93	0.000
Methanol to oil mole ratio	3	29.986	9.995	28.23	0.011
Time	3	40.033	13.344	37.69	0.007
Error	3	1.062	0.354		
Total	15	561.443			

S=0.5950, R^2^=0.998, Adj R^2^=0.995; where DF=degrees of freedom, Adj SS=adjusted sum of square, Adj MS=adjusted means of square, F=Probability distribution, p=probability.

In this example, ANOVA was employed to analyze only the effect of independent variables on biodiesel yield (%). The model was very suitable due to its p value, the coefficient of determination R^2^ (0.998) and the adjusted coefficient of determination adj R^2^ (0.995). The p‐values indicated that all the considered factors have statistical significance and, therefore, changes in the values of these factors contribute significantly to the response variable, due to p values being lower than 0.05. On the other hand, the larger F value and smaller p value were achieved for temperature, indicating this factor has a more significant effect on biodiesel yield. According to F values, the order of factors significance that affect biodiesel yield was temperature, followed by time, mole ratio, and catalyst amount. The same analysis based on F values could be used in other studies to determine the significance of different factors and their influence on evaluated responses variables, as has been reported by C. E. Aristizábal‐Alzate, et al.^[35]^ and Y. K. Chih, et al.^[4]^

Some reports present in their ANOVA the significance and effect of two‐ and three‐way interactions between factors over a response variable. For example, this case is shown by R. Rezaei, G. Moradi, S. Sharifnia,^[22]^ whose ANOVA result for catalyst deactivation is illustrated in Table 4. The process variables considered in this analysis were deposition time (X_1_), percentage of Cu/Ni surface area (X_2_), and reaction temperature (X_3_). They observed that all the independent factors (X_1_, X_2_ and X_3_) had a significant effect on Ni−Cu/Al_2_O_3_ deactivation, due to their p‐value being lower than 0.05. According to their F values, the most significant independent effect on the response variable was the reaction temperature, followed by deposition time and Cu/Ni surface area.

**Table 4:** ANOVA for catalyst deactivation.^[22]^

Source	Sum of squares	Mean square	F value	p Value
Model	83.51	9.28	80.11	<0.0001
X_1_	5.45	5.45	47.01	0.0010
X_2_	4.06	4.06	35.06	0.0020
X_3_	54.60	54.60	471.38	<0.0001
X_1_X_2_	0.022	0.022	0.19	0.6778
X_1_X_3_	0.12	0.12	1.06	0.3509
X_2_X_3_	0.000	0.000	0.000	1.000
$X_{1}^{2}$	0.86	0.86	7.45	0.0413
$X_{2}^{2}$	18.83	18.83	162.57	<0.0001
$X_{3}^{2}$	0.013	0.013	0.11	0.7552

Other important topic to be analyzed is the two‐way interaction influence (X_i_X_j_), which shows that there was no significant influence on the response variable and their effects were independent of catalyst deactivation (p‐value>0.05). Finally, the second‐order effect ($X_{i}^{2}$ ) evaluation showed that only $X_{2}^{2}$ has statistical significance. Although the second order effects did not have a phenomenological explanation, this result can be used to determine the inclusion of these effects in the regression model, used in optimization methods. Additionally, when independents, two‐way interactions and second order effects are included in ANOVA, the contribution to the variance of these effects is presented using the total F‐value for each group of effects.^[50]^ In accordance with Table 4, the sums of the F‐values for individual or independent factors (553.45) were relatively more significant in catalyst deactivation than two‐way interactions (1.25) and second order effects (170.13), due to individual effects contributed with 76.35 % of the variance. Therefore, independent changes in the variables considered in the ANOVA directly affected the deactivation response of the Ni−Cu/Al_2_O_3_ catalyst.

**Figure 3:**
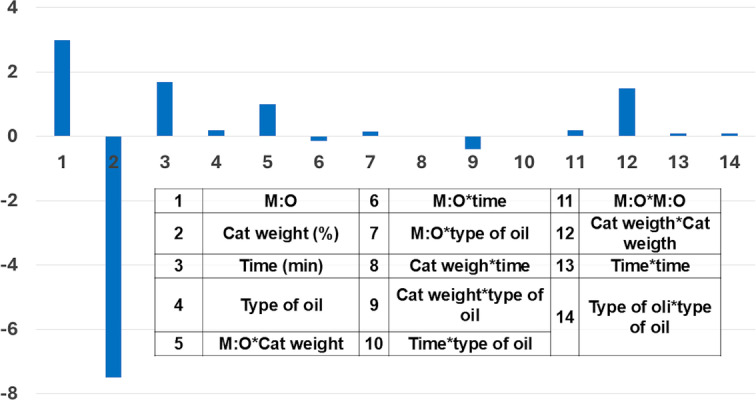
Pareto chart displaying the effects of various independent variables on the percentage of biodiesel yield. Adapted from N. S. El‐Gendy, S. F. Deriase.^[15]^

Another statistical tool is Pareto chart of the effects, which is employed to help and to understand in a visual way the interaction between the most relevant effects with the response variable.[^8^, ^15^] As an example, Pareto chart presented in Figure 3 highlights the primary effects and interactions among factors that predominantly affect the percentage of biodiesel yield in the work presented by N. S. El‐Gendy and S. F. Deriase.^[15]^

According to Figure 3, catalyst weight exhibited a significant negative impact on the percentage yield, indicating that an increase in catalyst loading leads to a decrease in yield. Conversely, the methanol to oil molar ratio (M : O) confirmed a positive effect, where higher ratios resulted in increased process yield. Nevertheless, the interaction between M : O molar ratio and reaction time displayed a marginal negative impact, suggesting that an increase in both variables simultaneously leads to a reduction in biodiesel yield percentage. In contrast, the interaction between M : O ratio and catalyst weight show a slight positive effect. Furthermore, reaction time appears to have a minor positive influence, whereas the type of oil factor demonstrates negligible impact on the percentage yield of biodiesel produced.

**Figure 4:**
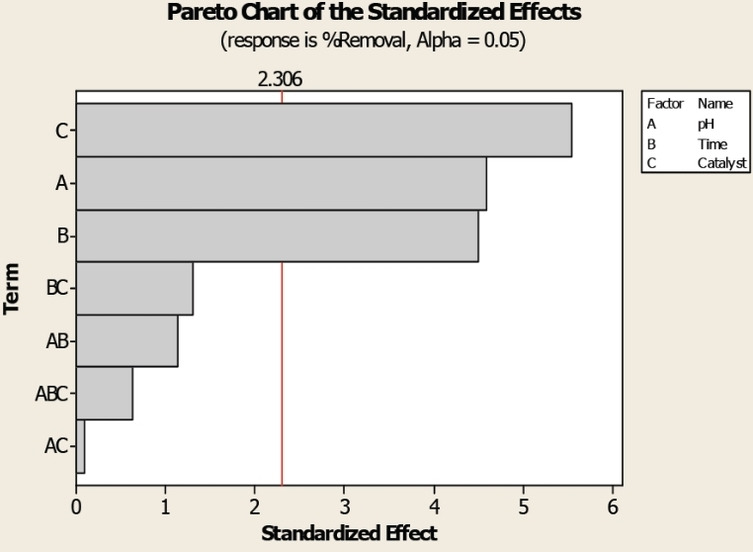
Pareto chart of the standardized effects for RY15 removal (%). Reproduced from Y. Asci, N. Ayas, E. A. Demirtas^[25]^ with permission of Elsevier.

Another way to present Pareto chart is with standardized effects, which can be used to see the relevance of independent, two‐way, and three‐way interaction effects on a response variable. Figure 4 shows a Pareto chart done by Y. Asci, et al.^[25]^

The primary effect plots shown in Figure 4 illustrates that catalyst weight (C) exhibited the most significant influence on RY15 removal, followed by pH (A), and contact time (B). Moreover, the two‐way and three‐way interaction effects did not show statistical significance in color removal, because these effects are smaller than the average value (2.306). Therefore, Pareto charts are an efficient visual way to qualitatively study and analyze the effects of different factors and their interaction with response variables. Unlike Pareto chart illustrated in Figure 3, which allows a clear identification of positive or negative impacts of factors on the response variable, the standardized effects representation (Figure 4) does not allow this distinction.

**Figure 5:**
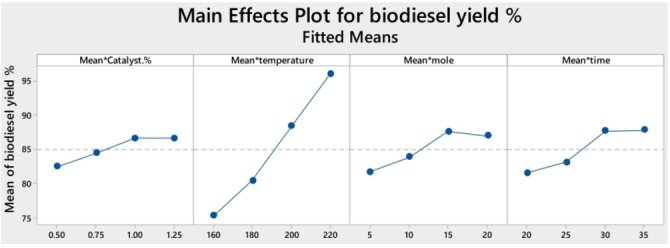
Effect of process parameters on biodiesel yield. Reproduced from M. N. Hossain, et al.^[48]^ with permission of MDPI.

Another type of graph is the “Main Effects Plot”. This graph contributes to establishing which of the levels considered for each factor or independent variable has the greatest significance in the response variable. In each factor plot, the maximum values of the response variable indicate the optimum condition for an individual factor. In addition, general trends of the factor's influence on the process can be determined.[^14^, ^48^] Furthermore, to obtain the optimal values of the factors that improve the response variable, mathematical models must be developed to achieve their optimization. However, each model obtained must be applied to its own experimental conditions; extrapolation of the data is not recommended. Figure 5 shows an example of these graphs for biodiesel yield by evaluating the influence of four factors: catalyst amount, reaction temperature, M : O ratio, and reaction time.

According to Figure 5, the highest biodiesel yield was achieved with a catalyst load of 1 wt %. However, beyond this catalyst amount, the biodiesel yield decreased slightly. This phenomenon could be attributed to the disruptive effects of excess catalyst in the reaction mixture, leading to diffusion problems.^[51]^ An increase in temperature was correlated with a rise in biodiesel yield due to the heightened frequency of molecular collisions during the reaction process.^[52]^ The optimum biodiesel yield was achieved at a M : O ratio of 15 : 1. However, upon exceeding this ratio, the biodiesel yield decreased. This decrease can be attributed to the promotion of the reverse reaction at higher M : O ratios.[^52^, ^53^] Finally, the data indicate a positive correlation between reaction time and biodiesel yield. It is noteworthy that there was a marked increase in biodiesel yield when the reaction time was extended from 25 to 30 minutes, and that an increase in the reaction time above 30 to 35 minutes did not show a significant change in biodiesel yield Although the main effects plot indicated that 220 °C was the optimal reaction temperature, they selected 200 °C to explore biodiesel production at a lower temperature. Therefore, the results of the main effects plot should be interpreted with caution, considering the main objectives of the research

The analysis of Pareto charts, main effects plots, and ANOVA allows researchers to compare and to validate the behavior of explanatory variables with findings in previous studies, as illustrated in previous paragraph. Another example is shown by Rezaei, G. Moradi, S. Sharifnia,^[22]^ where reaction temperature was the factor with the greatest influence on catalyst deactivation, due to it enhances coke formation by methane decomposition. This statement can be corroborated and compared with published research on the catalytic methane reforming.^[54]^

## 5 Response Surface Methodology

Response Surface Methodology (RSM) is a statistical technique used in several fields to optimize and understand the relationships between multiple variables. In catalysis, RSM helps researchers design experiments, analyze data, and predict optimal conditions for catalytic reactions. Recently, RSM has been widely applied to optimize and to analyze the interaction effects of independent variables in various chemical and biochemical processes.[^55^, ^56^, ^57^]

RSM is used as an advanced tool within experimental design techniques, and its main objective is to identify the optimal levels of variables that maximize the desired response.^[30]^ This technique has been adopted by several reports, due to its ability to offer closer and more accurate solutions to existing problems by considering higher order interactions and to identify the optimal conditions that will give optimal performance. RSM typically involves a variety of mathematical and statistical methods using empirical models developed through structured experimental designs.[^16^, ^39^] This involves using two factorial, linear, quadratic, or polynomial functions to represent the system under study, thus facilitating the exploration of experimental conditions towards optimization.^[24]^ Before employing RSM, it is imperative to select an appropriate experimental design to determine the number of experiments within the experimental region of the study.^[39]^

The most common analysis using RSM and reported by some authors[^10^, ^11^, ^40^, ^47^] is applied through a second‐order polynomial equation, which is an empirical model to correlate the response to the explanatory variables or factors. In the process involving independent variables X_i_, the association between the response variable Y and these variables can be approximated by considering all linear terms, linear‐to‐linear interaction terms, and square terms in the quadratic second‐order response model, as is described in Equation 1.

(1) $$Y=\beta_{o}+\sum_{i=1}^{k}\beta_{i}X_{i}+\sum_{i=1}^{k}\beta_{ii}X_{ii}^{2}+\sum_{i=1}^{k-1}\sum_{j=i+1}^{k}\beta_{ij}X_{i}X_{j}+\epsilon$$

where $\beta_{o}$ is the constant, $\beta_{i}$ is the slope or linear effect of the input factor X_i_, $\beta_{ij}$ the linear‐by‐linear interaction effect between the input factor X_i_ and X_j_, $\beta_{ii}\;$ is the quadratic effect of input factor X_i_ and ϵ is the random error.

Uncertainty in model predictions is predominantly influenced by two primary factors, sample size and model coefficients. A smaller sample size may introduce bias through increased variability, while an excessively large sample size may lead to numerical overflow and higher costs. Therefore, it is essential to carefully determine an optimal sample size to build statistical models with a high level of confidence. Furthermore, optimal conditions were generated by analyzing the model responses for each experiment or by solving the obtained mathematical model.[^15^, ^39^] The prediction models obtained must be solved with specialized software, as demonstrated by N. S. El‐Gendy, S. F. Deriase,^[15]^ who used an optimization software called Lingo. Table 5 shows some of the mathematical models obtained for several works that include this mathematical regression to optimize the effect on the response variable.

**Table 5:** RSM models for reported literature applied in heterogeneous catalyst research.

Model	Factor definition and considered levels	Optimum values for response variable and factors	Reference
%FAMEYield=-1975.08+46.51A+1.79B+41.97C $-\;0.008AB-0.003BC-0.105AC-0.33A^{2}-\;0.0023B^{2}\;$ $-1.089C^{2}$ R^2^=0.981, ϵ=2.19	A: Reaction temperature (°C). (60, 65 and 70) B: Reaction time (min). (180, 240 and 300) C: Methanol to oil molar ratio. (12, 15 and 18) The model uses the coded values for each level (−1, 0 and +1)	Optimum FAME yield (%)=94.90 % Reaction temperature=65 °C Methanol to oil molar ratio=15 Reaction time=240 min	C. S. Latchubugata, et a.l^[39]^
$\%\;Total\;chemical\;oxygen\;demand\;removal\;\left(TCOD\right)=$ 59.69-9.72A+5.61B+5.62C+13.31D $-3.04A^{2}-8.7B^{2}-4.29D^{2}+2.93BD$ R^2^=0.92	A: pH (2, 4, 6, 8 and 10) B: catalyst concentration (mg L^−1^). (0, 50, 150 and 200) C: reaction temperature (°C). (22.5, 30, 37,5, 45 and 52.5) D: reaction time (min). (30, 60, 90, 120 and 150) The model uses the coded values for each level (−1.5, −1, 0, 1 and 1.5)	Optimum TCOD remotion (%)=80.84 pH=4 Catalyst concentration (mg L^−1^)=150 Reaction temperature (°C)=45 Reaction time (min)=120	F. Shahrezaei, et al.^[24]^
$Y=54.717+2.884X_{1}-7.2601X_{2}+1,6297X_{3}+0.1929X_{4}$ $+0.9666X_{1}X_{2}-0.1185X_{1}X_{3}+0.07743X_{1}X_{4}-0.0307X_{2}X_{3}$ $-0,2973X_{2}X_{4}-0.0237X_{3}X_{4}+0.1253X_{1}^{2}+1.4547X_{2}^{2}$ $-0.0026X_{3}^{2}+1.0026X_{4}^{2}$ R^2^=0.9872	$X_{1}$ : methanol to oil molar ratio. (6, 9 and 12) $X_{2}:\;$ catalyst concentration (wt %). (3, 6 and 9) $X_{3}\;$ : Reaction time (min). (30, 60 and 120) $X_{4}$ : type of waste cooking oil. (WFSFO, WFCO and WFO) The model uses the coded values for each level (1, 2 and 3)	Optimum biodiesel Yield (wt %)=90.22 Methanol to oil molar ratio=9.15 Catalyst concentration (wt %)=7.73 Reaction time (min)=75 These results are independent of the type of WCO feedstock	N. S. El‐Gendy, S. F. Deriase^[15]^
$Y\;\left(\%\right)=8.65+1.08X^{1}+0.90X^{2}+\;0.80X^{3}$ $+0.66X^{1}X^{2}+0.66X^{1}X^{3}+0.50X^{2}X^{3}-\;3.36X^{12}$ $-2.73X^{2}-3.17X^{2}$ R^2^=0.99	$X_{1}$ : Pyrolysis reaction temperature (°C). (450, 500 and 550) $X_{2}:\;$ Catalyst to biomass mass ratio (C : B). (0.5 : 1.0, 1.0 : 1.0 and 1.5 : 1.0) $X_{3}\;$ : Nickel to Cerium mass ratio (Ni : Ce). (2 : 4, 3 : 3 and 4 : 2) The model uses the coded values for each level (−1, 0 and 1)	Optimum total C_6_‐C_8_ hydrocarbons in pyrolysis oil (%)=8.90 Pyrolysis reaction temperature (°C)=505 Catalyst to biomass mass ratio=1.1 : 1.0 Nickel to Cerium mass ratio=3.14 : 2.86	V. Balasundram, et al.^[12]^
$Y\;\left(\%\right)=-619.343+3.573X^{1}-0.006X^{12}+0.44X^{2}+1.766X^{3}-$ $0.057X^{2}-0.001X\mathrm{char}/0x2083\mathrm{not}\mathrm{implemented}-0.002X\mathrm{char}/0x2083\mathrm{not}\mathrm{implemented}$ R^2=^0.9915	$X_{1}$ : Reaction temperature (°C). (250, 300 and 350) $X_{2}:\;$ Treatment temperature (°C). (400, 450 and 500) $X_{3}\;$ : Treatment time (h). (3, 6 and 9)	The optimum yield of heptane isomers (%)=45.7 Reaction temperature (°C)=311 Treatment temperature (°C)=464 Treatment time (h)=6	N. A. A. Fatah, et al.^[32]^

Many of these studies also incorporated ANOVA, Pareto charts, and main effects plots, allowing for a focused analysis of the most influential factors. These tools qualitatively and quantitatively highlight which variables have significance within the experimental procedures and regression models. F and p values were used to check the significance of the corresponding coefficient.[^24^, ^48^] According to Table 5, the model obtained by F. Shahrezaei, et al.^[24]^ used this premise to suppress the coefficient terms for C^2^, AB, AC, AD, BC and CD to simplify the model. Although, C.S. Latchubugata, et a.l^[39]^ reported the coefficient for all effects. However, they mentioned the same premise to obtain a simpler model. On the other hand, the optimum values for factors presented by N.S. El‐Gendy, S.F. Deriase^[15]^ reported values between the considered levels for each explanatory variable. Therefore, it could be useful to evaluate this optimum in experimental conditions and to analyze the results.

To assess the model fit, the determination R^2^ is examined. For example, in N. S. El‐Gendy, S. F. Deriase,^[15]^ the R^2^ value of 0.9872 suggests that the model can account for 98.72 % of the variability. This implies that only 1.28 % of the total variations in equation remain unexplained by the model, which corroborates the goodness of fit and validates the adequacy of the regression model. Moreover, the close correspondence between the experimental and predicted values of the response variable further supports this conclusion. However, if the R^2^ and adjusted R^2^ values of the regression model are quite low, the inclusion of omitted explanatory variables, factors or the interactions between them, should be included to ensure the fit of the model regression.^[8]^

The sign of coefficient in the model ($\beta_{i},\beta_{ii}\;and\;\beta_{ij}$ ) helps to determine the performance of response variable. A coefficient variable with a positive sign denotes a congruent effect of the variable on the response, while a negative sign translates into an adverse effect.^[23]^ According to O. Ouled Ltaief, et al.,^[49]^ positive coefficients of factors in the model indicate their contributions to the enhancement of methyl orange, while, they suggest their negative impact when considering the improvements in the degradation rate. On the other hand, S. Monyanon, et al.^[8]^ found that the negative coefficient estimation of liquid flow rate in the regression equation indicates that a reduction in liquid feed rate results in elevated methanol conversion.

## 6 Synergistic Application of DOE, ANOVA and RSM in the Optimization of Heterogeneous Catalytic Processes

The combined application of DOE, ANOVA, and RSM provides a robust framework for optimizing processes in heterogeneous catalysis. These methodologies complement each other, and allow researchers to systematically explore reaction parameters, validate their importance, and optimize them for maximum catalytic efficiency.[^20^, ^58^]

However, simply identifying important factors is not enough to understand their influence on the response variables. Therefore, ANOVA plays an important role in validating the statistical significance of the factors identified by DOE, since by analyzing the experimental data, ANOVA ensures that the observed effects are statistically robust and not due to random variation.[^20^, ^35^, ^56^] This approach allows determining the reliability of the experimental conclusions based on the selected experiments and the statistical analysis of the variables. Once significant factors have been identified, RSM allows further optimization by modeling the relationships between variables, and predicting optimal conditions for catalytic performance.[^10^, ^12^] Therefore, the synergy between DOE, ANOVA, and RSM ensures a comprehensive approach to experimental design and optimization. DOE helps explore the relevant factors, ANOVA confirms their statistical significance, and RSM fine‐tunes the process to achieve optimal results. In heterogeneous catalysis, this integrated approach leads to more efficient use of resources, higher reaction yields, and improved catalyst performance, as evidenced by the studies reported throughout this manuscript.

## 7 Summary and Outlook

This minireview examines the application of DOE, ANOVA and RSM techniques in the field of heterogeneous catalysis, focusing on the catalyst development and processes. Heterogeneous catalysis plays a crucial role in several industrial sectors, including chemical, petrochemical, and environmental engineering, by facilitating crucial chemical transformations.[^6^, ^59^, ^60^]

The statistical approach works as a powerful tool to optimize catalyst performance and understand the relationships between process and response variables. This review examines the basic principles of these methods, highlighting their reliability in experiment design and data analysis. Furthermore, it reviews recent advances and innovative applications of these statistical techniques within heterogeneous catalysis processes, covering areas such as catalyst synthesis, detailed characterization, and studies on reaction kinetics. By elucidating the current state‐of‐the‐art methodologies and challenges, this minireview aims to provide valuable insights for researchers in the field of heterogeneous catalysis. Table 6 shows the most common data requirements, limitations, used software, and expected outcomes of DOE, ANOVA, and RSM. The information contained in this table is a summary obtained after analysing the works that appear in the references used in this manuscript.

**Table 6:** Comparative Analysis of Statistical Tools: Design of Experiments (DOE), Analysis of Variance (ANOVA), and Response Surface Methodology (RSM).

Statistical Tool	Data Requirements	Limitations	Expected outcomes	Used software
DOE	It requires a defined set of independent variables (factors) and response variables. Data collection is conducted under controlled experimental conditions, often using factorial or fractional factorial designs.	If it is not selected carefully, it can become resource‐intensive and time‐consuming, especially with many factors and interactions. Full factorial designs exponentially increase the number of experiments needed, potentially straining resources.	It helps quantify the effect of individual factors and their interactions, allowing for the optimization of complex processes. It is particularly useful in early‐stage experimental designs where the relationship between variables is not well known.	MATLAB Statistica Design Expert MINITAB SPSS
ANOVA	It requires continuous dependent variables and categorical independent variables. Data must meet assumptions of normality and homogeneity of variances for accurate results. Typically used to compare means across different groups of factors.	It assumes normal distribution of data and equal variance (homoscedasticity). It is sensitive to outliers and missing data. If assumptions are violated, the results can be misleading.	It identifies whether the differences between factors group means are statistically significant, making it ideal for comparing experimental results across multiple treatments or conditions.	
RSM	It requires quantitative data from experimental designs (e. g., factorial design, central composite, Box‐Behnken) to model the relationship between multiple independent variables and one or more dependent variables.	It requires careful model fitting; poor experimental design or inadequate data can lead to unreliable results. The complexity increases with the number of variables, and interpreting the interaction between variables can be challenging.	It enables the modeling and optimization of processes by exploring the relationship between factors and responses. It is especially useful for fine‐tuning experimental conditions to reach optimal performance.	

The integration of advanced statistical techniques and computational modeling holds great promise for advancing the application in the study and development of heterogeneous catalysis. Future research efforts could focus on the development of hybrid experimental‐computational frameworks, which combine the strengths of these statistically based methodologies with machine learning algorithms and molecular simulations to accurately interpret and extract information from this vast data set. This interdisciplinary approach would enable a more holistic understanding of the catalytic nature of some chemical reactions, facilitating the design and evaluation of different catalysts with improved performance and selectivity. Furthermore, there is a significant need to address key challenges such as optimization of multivariate systems and development of robust experimental protocols for complex catalytic processes. Recent advances in chemometrics, statistical learning, machine learning, and artificial intelligence have demonstrated significant utility in addressing this challenge.[^38^, ^44^]

Collaborative efforts between experimental, theoretical, and data scientists are essential to overcome these challenges and foster innovation in heterogeneous catalysis research. Finally, by leveraging synergies between the aforementioned statistical methodologies and emerging technologies, the discovery and development of sustainable catalytic solutions to address global energy issues, high value‐added chemical synthesis, and environmental challenges can be accelerated.

## Abbreviations

Adj MS: Adjusted Mean Squares
Adj R^2^
: Adjusted Coefficient of Determination
Adj SS: Adjusted Sum of Squares
ANOVA: Analysis of variance
BBD: Box‐Behnken Design
CCD: Central Composite Design
DF: Degrees of freedom
DOE: Design of experiments
F‐test: Fischer's Test
p‐value: Probability Value
R^2^
: Coefficient of Determination
RSM: Response surface methodology
S: Standard deviation
S/N: Signal‐to‐Noise Ratio

## Conflict of Interests

The authors declare no conflict of interest.

8

## Biographical Information

*Eva Castillejos López, an Associate Professor at UNED University, specializes in nanomaterials for catalysis. With a postdoctoral background at LCC‐CNRS, ICP‐CISC, and Assistant Professor at Complutense University, she has contributed to 20 research projects, including European, national, and regional. She has been active in the field of heterogeneous catalysis for processes such as environmental chemistry, CO_2_ conversion, or H_2_ chemical storage. Recognized for her expertise, Eva serves as the coordinator of the Environmental Sciences degree program at UNED University and received the Salvador de Madariaga grant at Cambridge University*.

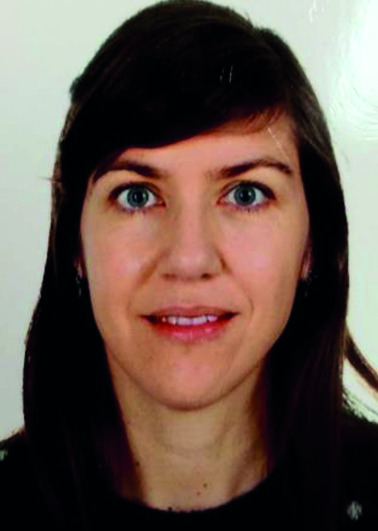

## Biographical Information

*Carlos E. Aristizábal‐Alzate received his B.S. in Chemical Engineering from the Universidad Nacional de Colombia (Medellín, Colombia) in 2012. Additionally, in 2020, he received his M.Sc. in Industrial Energy Management from the Instituto Tecnológico Metropolitano (Medellín, Colombia) and he is currently working as a Ph.D. engineering student at the same university. His primary research interests focus on simulation and data analysis about heterogeneous catalysis, harnessing and valorization of residual biomasses, biorefineries, carbon footprint calculation, and chemical processes in general*.

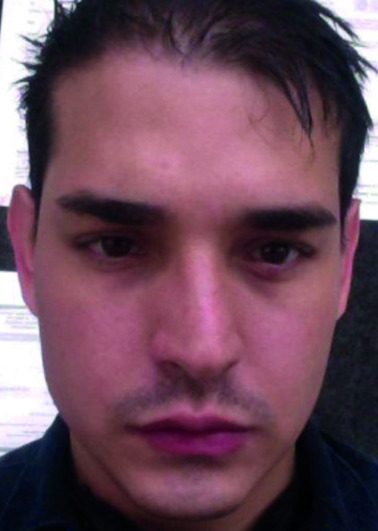

## Biographical Information

*Manuel Romero‐Sáez received his B.S. degree in chemistry from Universidad del País Vasco‐UPV/EHU (Spain) in 2006. He obtained his M.Sc. and Ph.D. in chemical process engineering and sustainable development from UPV/EHU in 2007 and 2012, respectively, and completed a postdoctoral degree in Department of Chemical Engineering and Biotechnology at University of Chile (Chile) between 2013 and 2016. He is currently working as assistant professor at Instituto Tecnológico Metropolitano (Medellín, Colombia). His primary research interests focus on energetic and environmental heterogeneous catalysis, including hydrogenation processes, such as CO_2_ methanation, and photocatalysis processes for the removal of different compounds in water, among others*.

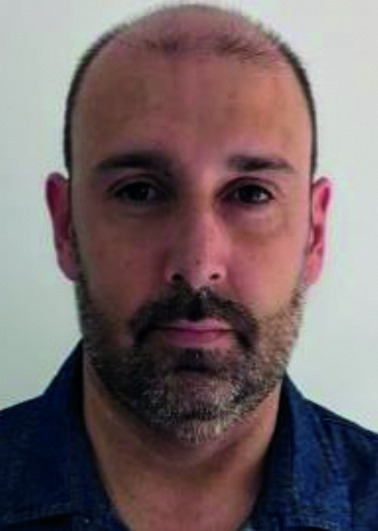

## Biographical Information

*Ana Belén Dongil, Teanured Scientist at Institute of Catalysis and Petrochemistry (Spanish National Research Council), specializes in heterogeneous catalysis. After receiving her PhD. in chemical engineering in 2011, she worked as researcher at ETH in Zürich and Concepción University (Chile) also as associated professor. Her research contribution includes more than 50 manuscripts, and she has lead 10 international and national research projects. She has been active in the field of heterogeneous catalysis for processes such as fine chemistry, CO_2_ capture and conversion and hydrogenation reactions applied to biomass valorization*.

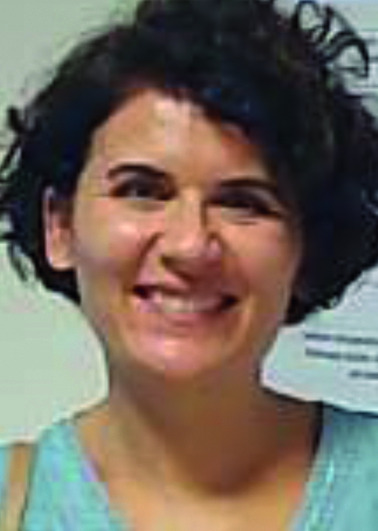

## Data Availability

Data sharing is not applicable to this article as no new data were created or analyzed in this study.
